# Association Between Prior Insurance and Health Service Utilization Among the Long-Term Uninsured in South Carolina

**DOI:** 10.1089/heq.2019.0014

**Published:** 2019-08-14

**Authors:** Lu Shi, Ellen C. Francis, Chaoling Feng, Xi Pan, Khoa Truong

**Affiliations:** ^1^ Department of Public Health Sciences, Clemson University, Clemson, South Carolina.; ^2^ IBM Watson Health, Truven Analytics, Bethesda, Maryland.; ^3^ Department of Sociology, Texas State University, San Marcos, Texas.

**Keywords:** long-term uninsured, health service utilization, prior insurance

## Abstract

**Purpose:** Strong evidence supports the relationship between health coverage and improved health status. Little is known about the lasting impact of prior health insurance on the prior insured's use of health services. We aimed to examine the association between prior insurance status and health service utilization (HSU) among the long-term uninsured (LTU) in South Carolina.

**Methods:** The current study used data from in-person interviews of the LTU collected in a 2014 cross-sectional South Carolina survey. Men and women between 18–64 years of age who reported not having health insurance for at least 24 months at the time of data collection were included. Propensity score analysis was used to examine the associations between prior insurance status and three outcome variables: (1) having a usual source of care, (2) HSU, and (3) delaying health care needs.

**Results:** Prior health insurance significantly predicted a greater likelihood of having a usual source of care (effect size: 9.2%, *p*=0.004) and having had at least one preventive visit during the past 2 years (effect size: 6.4%, *p*=0.035). Prior insurance coverage was positively associated with delayed health care utilization, but the result was not statistically significant (*p*=0.703).

**Conclusions:** Among the LTU, ever having insurance coverage was positively associated with having a usual source of care and HSU. The lasting impact of insurance coverage on HSU behavior extends beyond the period of insurance coverage, which provides a more comprehensive and deeper understanding of the long-term implications of national and local efforts in expanding insurance coverage.

## Introduction

Strong evidence supports the relationship between health insurance, health service utilization (HSU), and improved health status. Having a usual source of care is often the cornerstone of health care that facilitates prevention, continuity of care, and chronic disease management.^1–7^ Thus, health insurance and subsequent utilization are critical to maintaining health as it encompasses both preventive care and the treatment of illnesses.^8^ Previous research on the benefits of having a usual source of care^1,9–11^ and more recent findings on the benefits of patient centered medical homes,^12,13^ support the patient–provider relationship as being an access point for preventative health services.

The estimated rate of the uninsured population in South Carolina was 15% in 2014, placing South Carolina as the sixth highest rate of uninsured after the implementation of the Affordable Care Act (ACA). For some, lack of insurance represents a brief gap between policies, but the long-term uninsured (LTU) are a core part of the uninsured population, which in South Carolina, little is known about. Persons without health insurance for 24 months or longer are typically considered the LTU.^14,15^

Health insurance protects the insured against high and unexpected out-of-pocket costs and has the potential to ensure timely management of disease.^5^ The enactment of the 2010 Patient Protection and Affordable Care Act (PPACA), a comprehensive health care reform, including insurance expansion legislation, led to a steady decline in the uninsured rate from 17.1% in 2013 to 10.6% in 2016.^16^ Despite decreases in the rate of uninsured, frequently changing from an insured to an uninsured status and vice versa has been reported to encumber HSU, particularly among persons with lower socioeconomic status.^17^

Sustaining health coverage is an important factor for establishing and maintaining a usual source of care, and in both children and adult cohorts, obtaining health coverage has been associated with improvements in health and a usual source of care.^18,19^ It has been reported in a longitudinal study of health coverage that up to 35% of individuals who were previously insured lose their usual source of care once they become uninsured.^20^

The dynamic relationship between health insurance and HSU has been most frequently investigated among individuals who change from an uninsured status to an insured status,^8,21,22^ with the overall consensus supporting that health coverage increases access to care and benefits an individual's health. However, studies of HSU that include persons who never had insurance or have been uninsured for 24 months or longer are lacking. Considering the current political climate surrounding health care reform, and differential support for the repeal of Medicaid expansion, it is critical to understand the potential benefits of having prior insurance among the uninsured.

This article aimed to investigate the relationship between prior health insurance and HSU among those who had been uninsured for at least 24 months (LTU). Specifically, we examined the three following HSU outcomes: (1) having a usual source of care, (2) having a preventative care visit in the past 2 years, and (3) having delayed care utilization when ill during the past year.

## Methods

### Study population

The current study used in-person interview data collected between May 2014 and January 2015 from a South Carolina statewide survey of the LTU based on multistage sampling. The original study design was exploratory in nature—with the objective of understanding the characteristics of the LTU, why they remained uninsured, what was their current health status, and who provided or coordinated care for them. The survey took place a few months into the implementation of the individual mandate of the ACA. The details of the survey, including sampling method and data collection, are detailed elsewhere.^23^

**FIG. 1.**
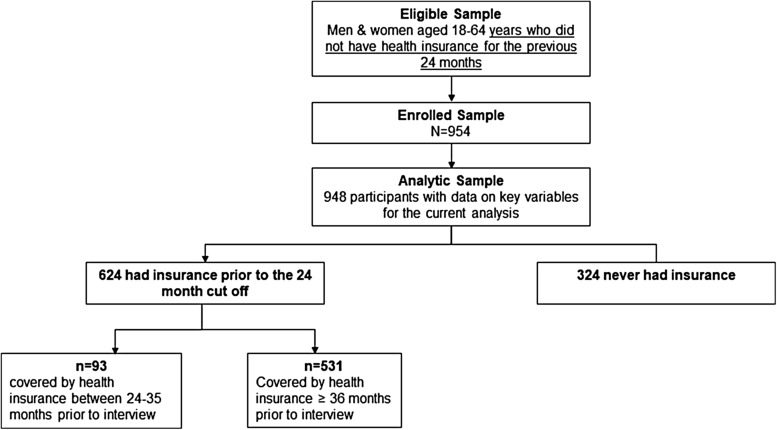
Diagram of participant selection for the current analysis. All participants reported not having health insurance in the 24 months before their interview date. Of these eligible participants, 948 reported data on key variables for our analysis. Among the analytic sample, 34.2% never had health insurance coverage.

South Carolina residents aged 18–64 who had been uninsured for 24 months or longer at the time of interview were eligible to participate in the survey. The survey consisted of questions relating to current health status, access to health care services, HSU, attempts to gain health coverage, and other social demographic questions. A total of 954 respondents participated in the survey. After excluding six participants due to missing responses, our final analytic sample included 948 respondents (Fig. 1). The study protocol and methods were approved by the Institutional Review Board of Clemson University.

### Measures

#### Predictor variable

In the current analysis, respondent self-reported prior health coverage (coded yes/no) was the main predictor.

#### HSU outcome variables

##### Usual source of care

Determination of having a usual source of care was based on a response of “yes” or “multiple” to the survey question “is there a particular doctor's office, clinic, minute clinic, health center, or other place that you usually go if you are sick or need advice about your health?,” and dichotomously coded as yes/no.

##### Preventative care visit in the past 2 years

Receipt of preventative care services was dichotomously coded as yes/no based on if a respondent reported a routine checkup within the past 2 years.

##### Postponement of health care

Postponing health care services was dichotomously coded as yes/no based on a respondent reporting postponing needed health care services for any reason (cost, logistical, or convenience).

#### Covariates

We explored the impact of age, gender, race, educational attainment, employment status, household income, self-reported health status, self-reported chronic condition, and respondent's zip code. Age was treated as a continuous variable; race/ethnicity was categorized as African American, white, and other. Educational attainment was operationalized as a nine-level categorical variable ranging from “no formal education” to “advanced degree.” Employment status was defined as full-time, part-time, or retired. Self-reported health status was coded as five categories ranging from “excellent” to “poor.” Participants who reported that they had been told they had a serious long-lasting health problem such as diabetes/high blood sugar, high blood pressure, or high cholesterol were categorized as having a self-reported chronic condition.

### Analysis

Statistical differences in respondent characteristics based on prior insurance status were examined by chi-square for categorical variables and *t*-tests for continuous variables. More than 20% of data were missing for household income and so it was not included in any multivariable analyses. Propensity score method was used to investigate the association between prior insurance and HSU, while accounting for differences in respondent characteristics. Propensity score methods can be used in observational studies to estimate the effect of an exposure while accounting for other factors that predict the likelihood of receiving the exposure and the outcome of interest.

It has been argued and shown that the use of a propensity score approach is an improvement over generalized linear models because of the creation of a more suitable comparison group and, as such, minimizes the chance of biased estimates.^24^ The suitability of the comparison group is determined by minimizing differences in participant characteristics that would predict, or be correlated, with the likelihood of them receiving treatment (exposed to having prior insurance). In the current study, respondent characteristics that were significantly associated with prior insurance (our exposure variable), indicating that some individual factors presumed to be predictive of having prior insurance were not balanced between those who did, and those who did not, report having prior insurance. As such, propensity scores were estimated using logistic regression to predict the likelihood of having prior insurance based on respondent characteristics of age, gender, race, educational attainment, and employment status.

In the current analysis, we used propensity score stratification, which is one of a variety of propensity score methodologies. This particular propensity score method stratifies the entire sample into quantiles, with respondents matched on the probability of receiving treatment (prior insurance), so that within each stratum the likelihood of being exposed is essentially random.^25,26^ Within each stratum the impact of the exposure on the outcome (HSU) is estimated while controlling for confounding respondent characteristics. The overall difference is computed as a weighted average of differences from each stratum, with weights based upon the frequency distribution of exposure across the stratum.^27^

To increase the robustness of the more novel propensity score method, we conducted sensitive analyses using logistic regression to estimate the odds ratio (OR) and 95% confidence intervals (CIs) for the association between prior insurance and HSU. We examined three model specifications: unadjusted, adjusted for race/ethnicity, education, employment, and gender, and further adjusted for self-reported health status and chronic conditions. To address potential bias in our estimates we additionally explored the impact of controlling for respondent zip code, which would account for zip code level factors that might be confounders. All analyses were conducted using Stata 11 (Stata Corp, College Station, TX).

## Results

Table 1 describes respondent sociodemographic characteristics of the LTU, as well as their HSU. The mean (standard deviation) age was 42.0 (12.7), 58.4% were women, 53.3% of respondents completed high school, 65.8% reported having prior insurance, and of the respondents who reported annual household income (*n*=752), 44.4% had an annual income that was <$10,000. Significant differences in respondent characteristics were observed between those with prior insurance compared to those without. For example, women were more likely to report prior insurance coverage than males (63.1% vs. 49.1%, *p*=0.0001). For participants who reported ever having health insurance (*n*=624), 14.1% reported coverage 24–25 months prior, and 85.1% reported that they had coverage 36 months prior or more. Among the respondents, 74.3% (*n*=702) had a usual source of care, 29.7% (*n*=281) reported having a preventative care visit in the past 2 years, and 66.7% (*n*=631) reported delaying needed health care in the past year.

**Table 1.** Characteristics of Uninsured (*n*=948)

Characteristics	*n* (%)	Prior coverage (*n*=624)	No prior coverage (*n*=324)	*p*
Age, years				0.924
Mean (standard deviation)	42 (12.7)	42.0 (12.8)	42.0 (12.5)	
Race, %				<0.0001
Black	676 (71.9)	441 (71.0)	235 (73.7)	
White	166 (17.7)	138 (22.2)	28 (8.8)	
Other	98 (10.4)	42 (6.8)	56 (17.6)	
Gender, %				<0.0001
Male				
Female	549 (58.4)	391 (63.1)	158 (49.4)	
Education, %				<0.0001
Did not complete middle school	46 (5.0)	9 (1.5)	37 (11.7)	
Completed middle school	269 (28.9)	157 (25.6)	112 (35.3)	
Completed high school	496 (53.3)	344 (56.1)	152 (48.0)	
Vocational school or more	119 (12.8)	103 (16.8)	16 (5.1)	
Employed				0.368
Full-time	76 (8.1)	55 (8.9)	21 (6.6)	
Part-time	863 (91.8)	565 (91.0)	298 (93.4)	
Retired	1 (0.1)	1 (0.2)	0 (0.0)	
Household income				0.001
<$10,000	334 (44.4)	226 (43.5)	108 (20.8)	
$10,000−$24,999	291 (38.7)	200 (38.5)	91 (17.5)	
$25,000−$49,999	91 (12.1)	67 (12.9)	24 (4.6)	
$50,000−$74,999	4 (0.5)	2 (0.4)	2 (0.4)	
>$75,000	32 (4.3)	25 (4.8)	7 (4.8)	
Time since last health insurance coverage^a^				NA
24–35 months	93 (14.9)	93 (14.9)	NA	
≥36 months	531 (85.1)	531 (85.1)	NA	
Health care utilization variables				
Had a usual source of care	702 (74.3)	483 (77.5)	216 (68.0)	0.002
Had a preventive visit during the past 2 years	281 (29.7)	119 (31.9)	82 (25.5)	0.039
Delayed needed care during the past 12 months	631 (66.7)	417 (66.9)	214 (66.5)	0.883

Not all respondents completed all sociodemographic questions.

^a^ For participants who never had health insurance coverage, time since last health coverage was NA.

NA, not applicable.

Supplementary Table S1 compares our sample characteristics with that estimated from the uninsured subsample in the South Carolina Behavioral Risk Factor Surveillance System (BRFSS) and Medical Expenditure Panel Survey (MEPS). All the other characteristics of uninsured residents in South Carolina were based on analysis of the uninsured resident sample from the 2015 BRFSS. The characteristics of our sample were not distinctively different from the BRFSS uninsured subsample in terms of health status, age, and gender. Our convenience sample of the LTU was different from a subset of the South Carolina uninsured population in terms of racial distribution and household income. Specifically, African Americans and low-income residents were of higher proportion in our LTU sample compared with the uninsured subset in BRFSS and MEPS.

The propensity score stratification demonstrated that having prior insurance significantly predicted greater likelihood of having a usual source of care (*p*=0.004). On average, a greater proportion of respondents with prior insurance reported having a usual source of care (9.2% higher, standard error [SE]=0.03). Similarly, on average a greater proportion of respondents with prior insurance reported having at least one preventive visit during the past 2 years (6.4% higher, SE=0.03, *p*=0.035) (Table 2). Although the relationship was positive, having had prior health insurance did not significantly predict the probability of delaying health care when needed (*p*=0.703). Furthermore, having prior health insurance was associated with gender, education, and race/ethnicity. Specially, women were more likely to have had prior insurance (*p*<0.001), people with higher educational attainment (*p*<0.001), and white respondents were more likely to have had prior insurance (*p*<0.001).

**Table 2.** Association Between Prior Insurance and Health Service Utilization

Health service utilization	ATT (SE)	*t*	*p*
Had a usual source of care	0.092 (0.031)^a^	3.08	0.003
Had a preventive visit during the past 2 years	0.064 (0.031)^a^	2.10	0.036
Delayed needed care during the past year	0.006 (0.032)	0.20	0.934

*n*=948. Data are presented as weighted average percentage difference (ATT) between persons with prior insurance compared to persons without and SE of percentage.

^a^ Statistically significant at 95% CI.

ATT, average treatment effect on the treated; SE, standard error.

In the unadjusted logistic regression models, respondents who reported prior insurance had an increased probability of having a usual source of care compared to those without prior insurance (OR=1.62, 95% CI: 1.18–2.14) (Table 3). After adjusting for potential confounders, prior insurance remained significantly associated with having a usual source of care (OR=1.61, 95% CI: 1.15–2.25). There was a significant positive association between prior insurance and having a preventative visit during the past 2 years (OR=1.37, 95% CI: 1.02–1.85), but these results became insignificant in the two adjusted model specifications (OR=1.32, 95% CI: 0.95–1.83; OR=1.25, 95% CI: 0.90–1.76). There was no significant association between prior insurance and delaying needed health care in all three model specifications (unadjusted model: OR=1.02, 95% CI: 0.77–1.36). The estimated associations were similar when accounting for zip code level factors (Supplementary Table S2).

**Table 3.** Associations Between Prior Insurance Coverage and Health Service Utilization

Health service utilization	Unadjusted	Model 1^a^	Model 2^b^
Had a usual source of care	1.62 (1.20–2.19)^c^	1.63 (1.17–2.26)^c^	1.61 (1.15–2.25)^c^
Had a preventive visit during the past 2 years	1.37 (1.02–1.85)^c^	1.32 (0.95–1.83)	1.25 (0.90–1.76)
Delayed needed care during the past year	1.02 (0.77–1.36)	0.93 (0.68–1.28)	0.96 (0.70–1.32)

*n*=948. Data are presented as odds ratios and 95% CI. Regression estimates are represented.

^a^ Adjusted models included race, education, employment, and gender.

^b^ Adjusted models included race, education, employment, gender, self-reported health status, and self-reported chronic conditions.

^c^ Statistically significant at 95% CI.

## Discussion

The current study used primary data collected from the LTU, a hard-to-reach population in the state of South Carolina, and is among the first to demonstrate the necessity of evaluating the benefit of health insurance on long-term HSU outcomes among the LTU. Results of the study provide a new perspective and add to our comprehensive understanding of the long-term implications of national and local efforts in expanding insurance coverage. Our finding of a positive association between prior insurance coverage and having a usual source of care, as well as receiving preventative care, is consistent with the objectives of health insurance—namely to facilitate access and receive preventative care services, while also protecting against the financial burden of a catastrophic event.^28^ Although our study was not designed to uncover the mechanism by which prior insurance influences HSU after becoming uninsured, we hypothesize that while insured, the barriers to establishing a usual source of care and receiving preventative services are reduced and that these effects are maintained after the loss of insurance coverage.

The capacity to obtain, process, understand basic health information, and communicate effectively to make informed health decisions may be one of the ancillary benefits from ever having had health insurance. In a study examining the association of health insurance change (gain or loss of coverage) and changes in preventive care and health behaviors, the researchers found a positive association with change in coverage and use of preventative services.^28^ Although they found that loss of coverage was associated with a reduced likelihood of obtaining preventive services, they only examined the year following health insurance change. This latter factor may be an important distinction as not all preventative services are suggested annually.

Our finding that having a usual source of care was associated with prior insurance coverage may help explain the pathway by which prior insurance facilitates HSU among the LTU. In a study examining the interaction between insurance coverage, having a usual source of care, and use of health services, they reported that having insurance, or having a usual source of care but no insurance, was associated with intermediate use of preventative services.^29^ The uniqueness of our data and population makes direct comparisons with previous studies difficult, but our findings do align with the notion that health insurance coverage has a positive impact on HSU. Efforts to understand HSU, and in particular the use of preventive services among the uninsured, may find our results useful in anticipating changes in utilizations. Furthermore, the utilization of health services while covered by health insurance could benefit the insured beyond the coverage duration.

In our sensitivity analysis, the unadjusted logistic regression models closely aligned with our findings when propensity score methods were used. It is important to recognize the difference in the estimated effects (what it is and how much it is) between the propensity score and logistic regression method. The average treatment effect (ATE), estimated with logistic regression in terms of ORs, represents the impact of prior insurance on HSU and gave the same weight to all individuals in the sample, including those who are highly unlikely to ever have had insurance. Conversely, the ATE among the treated, estimated with propensity score stratification, represents the likelihood of HSU based on prior insurance, but giving higher weight to respondents who are likely to have had prior insurance (even if in fact they never had). This latter distinction represents the improved comparison group obtained when using propensity score methods.^30,31^

While the two different methods resulted in estimates that were in a similar direction, the lack of congruency in statistical significance may be explained by how each method accounts for respondents who are dissimilar. Specifically, propensity score stratification uses weights to diminish the impact of outliers, or unwell matched respondents, while logistic regression treats every observation equally. As such, in logistic regression outliers may make greater contribution to the effect size and, thus, the statistical significance. Furthermore, if we reduced the CI from 95% to 90% for both the logistic regression and propensity score method, the results would be similar in terms of statistical significance.

Overall, our study fills a gap by examining the long-term impact of prior health insurance on HSU among a population that has been without insurance coverage for two or more years. The analysis came from our own primary data collection, shedding light on the health and health care of a hard to reach population. Despite the observed benefits of prior insurance among the LTU, more than two thirds of our respondents reported delaying needed health care, and less than one third reported receiving preventative care within the past 2 years. Delaying needed care and forgoing preventative care could later become a major expenditure for Medicaid and Medicare,^17^ with increased costs and worse prognoses. Delaying care fits neither the equity principle nor the efficiency principle in public finance. Therefore, it is important to explore what might be significant predictors of improving HSU behavior.

The current study also has limitations. First, the small sample does not provide enough statistical power to further examine characteristic associations and patterns. As such, some residual confounding may still be possible and relevant unobservable confounding differences may still exist between groups. Second, although some characteristics of our sample were similar to the general population of uninsured adults in South Carolina, our findings may not generalize to persons of higher income and different racial backgrounds. Third, given the limited scope of our survey, it is not possible to estimate the number of the LTU in South Carolina. In fact, estimates of the LTU are very limited.

The MEPS defines three types of uninsured persons as follows: (1) uninsured for at least a full year; (2) uninsured for a given point in time; and (3) uninsured for a period of time during the course of a year. Using the first definition—closest to our definition of the LTU, it was estimated that the percentage of uninsured nonelderly adults in the United States declined from 11.7% in 2015 (22.4 million adults) to 10.8% in 2016 (20.6 million adults). This decline however differs between Medicaid expansion states and nonexpansion states (including South Carolina). For Medicaid expansion states, the rate declined from 9.0% to 8.2%, but there was no significant change in nonexpansion states.^32^

Despite these limitations, this present analysis reveals that among the LTU, prior health coverage was associated with a greater likelihood of engaging in HSU, which may be related to knowledge and behavior patterns gained while insured. Future studies with larger sample sizes, longitudinal designs, and more comprehensive information on clinical and sociodemographic characteristics are needed to further our understanding on this issue.

## Health Equity Implications

Health insurance and subsequent utilization are critical to maintaining health as it encompasses both preventive care and the treatment of illnesses. Our study provided evidence of an indirect benefit of previous insurance coverage on HSU among the people who had not been insured for over 2 years. Expanding insurance coverage among the low-income population might have the unexpected benefit of encouraging them to seek necessary care, a behavior that might sustain even after the insurance coverage expires.

## Supplementary Material

Supplemental data

## Abbreviations Used

ACA: Affordable Care Act
ATE: average treatment effect
ATT: ATE on the treated
BRFSS: Behavioral Risk Factor Surveillance System
CI: confidence level
HSU: health service utilization
LTU: long-term uninsured
MEPS: Medical Expenditure Panel Survey
NA: not applicable
OR: odds ratio
PPACA: Patient Protection and Affordable Care Act
SE: standard error
