# Supplementary material for: Association Between Prior Insurance and Health Service Utilization Among the Long-Term Uninsured in South Carolina
Source: Health Equity. 2019 Aug 14;3(1):409–16. doi: 10.1089/heq.2019.0014 (PMC6705444; doi:10.1089/heq.2019.0014)
Supplement: Supplemental data [file Supp_TableS1-S2.pdf]

Supplementary Data

Supplementary Table S1. Long-Term Uninsured Sample Characteristic Comparisons with BRFSS and MEPS Uninsured Subsamples

Characteristics	Long-term uninsured ($n = 948$)	BRFSS uninsured ($n = 1088$)
Self-reported health status		
Excellent/very good (%)	27.00	38.14
Good (%)	30.77	36.40
Fair (%)	32.67	16.00
Poor (%)	9.48	9.19
Age, years (mean)	42.00	47.93
Race, %		
Black	71.9	33.46
White	17.7	52.67
Latino	6.42	6.62
Other	3.58	7.25
Gender (female %)	58.4	50.09
Household income (mean)	$13,626	$39,830

In the BRFSS uninsured sample column, all the characteristics of long-term uninsured residents in South Carolina are based on analysis of the 2015 BRFSS sample; age and household income are recalculated based on the income and age categories defined in the BRFSS dataset.

BRFSS, Behavioral Risk Factor Surveillance System; MEPS, Medical Expenditure Panel Survey.

Supplementary Table S2. Associations Between Prior Insurance Coverage and Health Service Utilization Controlling for Respondent Zip Code

Health service utilization	Unadjusted	Adjusted
Had a usual source of care	1.74 (1.25–2.41)[a]	1.71 (1.20–2.45)[a]
Had a preventive visit during the past 2 years	1.20 (0.88–1.65)	1.10 (0.78–1.51)
Delayed needed care during the past year	0.99 (0.73–1.35)	0.92 (0.66–1.28)

$n = 948$. Data are presented as odds ratios and 95% CI.

Adjusted models included race, education, employment, gender, self-reported health status, self-reported chronic conditions, and respondent zip code.

[a]Statistically significant at 95% CI.

CI, confidence level.
